# Monitoring inequality: an emerging priority for health post-2015

**DOI:** 10.2471/BLT.15.162081

**Published:** 2015-09-01

**Authors:** Ahmad Reza Hosseinpoor, Nicole Bergen, Veronica Magar

**Affiliations:** a World Health Organization, 20 Avenue Appia, CH-1211 Geneva 27, Switzerland.

The Millennium Development Goals focused on poverty and development and reducing inequalities between countries.^1^ Progress was monitored through national averages without adequate attention to within-country inequality. The post-2015 sustainable development goals (SDG) stress “leaving no one behind” – with goal 10 specifically calling for the reduction of inequality, within and among countries.^2^

Monitoring of inequalities within countries focuses on indicators and dimensions of inequality that are particularly relevant to each country. Drawing upon the outputs of within-country inequality monitoring, policies can be tailored to be maximally effective in reducing inequalities.^3^ At the same time, having comparable disaggregated data across countries is important to track within-country inequality at a regional or global level. One of the SDG targets specifically addresses the importance of disaggregated data, calling on countries to increase “…the availability of high-quality, timely and reliable data disaggregated by income, gender, age, race, ethnicity, migratory status, disability, geographic location and other characteristics relevant in national contexts.”^2^

Such disaggregated data are vital to identify where and why inequalities exist and ensure that policies, programmes and practices are successful in reaching the most vulnerable. Many countries have made major progress in monitoring health inequalities through household surveys such as Demographic and Health Surveys. However, currently, many basic indicators of health and well-being are not consistently available in a form that permits disaggregation by sociodemographic factors and subnational regions. For instance, relatively few studies have provided the global situation of within-country inequality in risk factors of noncommunicable diseases by socioeconomic status.^4^^,^^5^

Household health surveys now need to be extended to include more emphasis on noncommunicable diseases and injuries. Investments should be made across different data sources, including birth and death registration (with cause of death), health facility and community information systems and administrative data on health infrastructure, workforce and financing. Countries should develop technical capacity to conduct health inequality analysis and establish reporting practices that effectively communicate clear messages facilitating action.

Global standards are now required to enable better international comparisons of within-country health inequality. A coordinated effort to support standardized data collection, analysis and reporting in all countries will strengthen global and regional monitoring of inequality in health. The World Health Organization (WHO) Health Equity Monitor contains comparable disaggregated data from 94 countries on the topic of reproductive, maternal, newborn and child health.^6^ Using these data, a flagship report was developed to showcase best practices in reporting inequalities in low- and middle-income countries.^7^ Expanding such activities to cover other health topics and more countries would enable wider adoption of health inequality monitoring. This would serve to increase the accountability of health systems, and also other sectors whose actions have an impact on population health.

Goal 3 of the SDGs is “to ensure healthy lives and promote well-being for all at all ages.”^2^ Thus, all health-related SDG targets necessitate a pro-equity approach that promotes accelerated progress among the disadvantaged to close within-country gaps. The endorsement of universal health coverage as an SDG target demonstrates a commitment to equity, aiming to ensure that everyone who needs health services is able to get them without undue financial hardship. The progressive realization of universal health coverage seeks accelerated gains by disadvantaged populations, thereby narrowing coverage gaps and improving the health of the broader population.^8^^–^^10^ In a similar spirit, equity should be a focus of other health-related sustainable development targets.

A multitude of factors underlie successful health inequality monitoring systems: political will, financial resources, popular support, advocacy and technical expertise. Strengthening health inequality monitoring requires the engagement of diverse partners, including ministries of health, national statistical offices and other relevant sectors of governments, United Nations agencies, funding agencies, academic institutions, civil society organizations and the private sector. Building a multidisciplinary network of experts in the area of health inequality monitoring with diverse strengths and perspectives will foster the development of expertise to tackle health inequity.

The post-2015 era presents an opportunity for WHO and its partners to strengthen health inequality monitoring across all health topics at global, national and subnational levels. An equity-based approach to improving population health will move the world closer to the ideal of healthy lives and well-being for all.
